# Acupuncture to Improve Symptoms for Stable Angina: Protocol for a Randomized Controlled Trial

**DOI:** 10.2196/14705

**Published:** 2019-07-29

**Authors:** Judith Schlaeger, Hui Yan Cai, Alana D Steffen, Veronica Angulo, Adhir R Shroff, Joan E Briller, Debra Hoppensteadt, Glorieuse Uwizeye, Heather A Pauls, Miho Takayama, Hiroyoshi Yajima, Nobuari Takakura, Holli A DeVon

**Affiliations:** 1 Department of Women, Children and Family Health Science College of Nursing University of Illinois at Chicago Chicago, IL United States; 2 Department of Acupuncture and Oriental Medicine National University of Health Sciences Lagrange, IL United States; 3 Department of Health Systems Science College of Nursing University of Illinois at Chicago Chicago, IL United States; 4 Department of Cardiology College of Medicine University of Illinois at Chicago Chicago, IL United States; 5 Department of Pathology and Pharmacology Loyola University Medical Center Maywood, IL United States; 6 Department of Biobehavioral Health Science College of Nursing University of Illinois at Chicago Chicago, IL United States; 7 Office of Research Facilitation College of Nursing University of Illinois at Chicago Chicago, IL United States; 8 Department of Acupuncture and Moxibustion Tokyo Ariake University of Medical and Health Sciences Tokyo Japan

**Keywords:** acupuncture, stable angina, ischemic heart disease, complementary medicine

## Abstract

**Background:**

Acupuncture has demonstrated physiologic analgesic effects in Chinese patients with stable angina. One proposed mechanism of action for these analgesic effects is the downregulation of M1 macrophages, interleukin 1 beta, interleukin-6, interleukin-18, and tumor necrosis factor alpha.

**Objective:**

This study aims to test a 10-session, 5-week acupuncture treatment protocol as a complementary therapy for symptoms of stable angina for American patients, who vary from Chinese patients in health care systems and other salient variables.

**Methods:**

We are conducting a randomized controlled trial (RCT) of 69 adults (35 assigned to initial acupuncture and 34 to an attention control condition) with a medically confirmed diagnosis of stable angina, whose pain and associated symptoms have not been controlled *to their satisfaction* with guideline-directed medical management. Participants in the experimental group will receive a standardized traditional Chinese medicine point prescription. The attention control group will view non–pain-related health education videos over 5 weeks equal to the 10 hours of treatment for the acupuncture group. Participants will complete the McGill Pain Questionnaire and the Seattle Angina Questionnaire-7, as well as have inflammatory cytokines measured at baseline and study completion. The primary outcomes are anginal pain and quality of life.

**Results:**

This study has been funded over 2 years by the National Institutes of Health, National Institute for Nursing Research. We are currently recruiting and expect to have initial results by December 2020.

**Conclusions:**

We will generate data on feasibility, acceptability, effect sizes, and protocol revisions for a future fully powered RCT of the protocol. Findings will help determine if patients with persistent ischemic symptoms experience a proinflammatory state and hyperalgesia caused by multiple neural and immune processes not always relieved with medication.

**International Registered Report Identifier (IRRID):**

DERR1-10.2196/14705

## Introduction

### Background

The significance of managing the symptoms of stable angina is critical, given that stable angina increases the risk of acute coronary syndrome for women and mortality for men [1,2]. Camm et al [3] found that one-third of patients with stable angina have suboptimal management of their pain. Guideline-directed therapy for stable angina includes nitrates, beta-blockers, calcium channel blockers, and angiotensin-converting enzyme inhibitors [4]. These drugs can have side effects such as headache, flushing, fatigue, and cough, which can lead to nonadherence and reduced symptom control [5]. Acupuncture, if effective, may help control angina pain for patients nonadherent to medications because of debilitating side effects.

Acupuncture has demonstrated physiologic analgesic effects. It regulates the autonomic nervous system and reduces sympathetic stimulation to the heart and vasculature by modulating the midbrain, releasing endorphins and dynorphins, with a resultant decrease in production of norepinephrine and epinephrine [6-12]. These processes impact the cardiovascular system by reducing blood pressure, heart rate, and arrhythmias [13] centrally mediated in the brainstem [12]. Acupuncture may help relieve pain by activating mu opioid receptors, which decreases pain [6], increasing serum beta endorphins [7] and downregulating M1 macrophages, interleukin (IL) 1 beta, IL-6, ILin-18, and tumor necrosis factor (TNF) alpha [14], which reduces inflammation [15]. The mechanism of action is stimulation of a neuronal circuit that detects inflammatory mediators and releases dopamine, inhibiting release of inflammatory cytokines [15]. No studies have directly examined the physiologic effects of acupuncture on the cardiovascular system for the treatment of angina.

In traditional Chinese medicine (TCM), *qi* is the vital energy flowing within and surrounding the body [16]. Disorders of *qi* or blood can result in pain, and *qi* can be deficient or in excess (stagnant or obstructed). The channels through which *q* *i* and blood flow in the body are called meridians [17]. Acupuncture needles are inserted into acupuncture points, which access the meridians and promote the circulation of *qi* and blood [18]. Deficient *qi* and/or blood is strengthened (tonified); excess qi and/or blood is moved to reduce stagnation or obstruction [19,20]. Thus, the body becomes balanced, and pain and other symptoms are reduced [18,19]. In the TCM model, it is theorized that the pain, shortness of breath, and fatigue of stable angina result when there is an excess or a deficiency in *qi* and/or blood in the meridians that flow through and around the heart [20-22].

### Objectives

The scientific premise of our study is that patients with persistent ischemic symptoms experience a proinflammatory state and hyperalgesia caused by multiple neural and immune processes not always relieved with medication [15]. The aims of our study are to test the feasibility of a 10-session, 5-week acupuncture treatment protocol as a *complementary* therapy for symptoms of stable angina in *American* patients and to estimate effect sizes for change from pre- to postintervention scores for pain/symptoms, inflammatory cytokines, functional status, and health-related quality of life (HRQoL) between the acupuncture and control groups. We hypothesize that participants with stable angina will see a reduction in pain following acupuncture [8].

## Methods

### Study Design

This randomized attention-control feasibility study will be conducted at an academic medical center in the Midwest. This design will facilitate the calculation of effect sizes for within- and between-group differences in pain and symptoms. The design is more appropriate than either *placebo* or *sham* acupuncture interventions at this stage of investigation because the protocol has not been tested in an American population of patients with stable angina. A placebo control design is not possible because neither the clinician nor participant can be blinded to the acupuncture treatment without the use of double-blind acupuncture needles. Currently, double-blind needles are being produced by hand, tested in small studies, and are not yet commercially available [23,24]. Sham acupuncture, whereby needles are inserted into nonacupuncture points on the body, is inferior to randomized control trial (RCT) with attention control design because needle punctures anywhere on the skin may be considered an *ashi* point (ie, an active acupuncture point that may have therapeutic effects) [25,26]. Following consent and upon enrollment in the study, participants will be randomized 1:1 to the acupuncture or attention control group using a permuted block approach, stratified by biological sex. This study was approved by the University of Illinois at Chicago Institutional Review Board (UIC IRB).

### Sample and Setting

**Figure 1:**
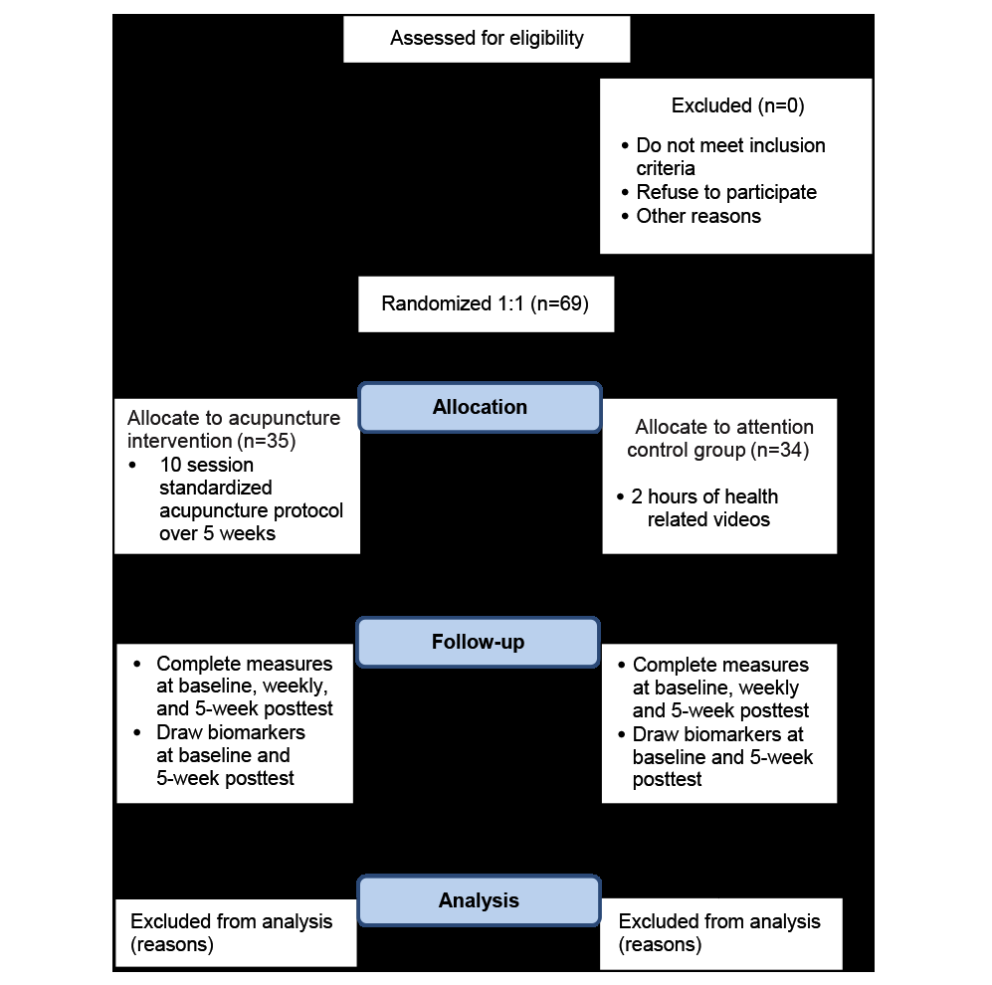
CONSORT (Consolidated Standards of Reporting Trials) flow chart.

A total of 69 patients with a confirmed diagnosis of stable angina will be included. Only those patients with Canadian Cardiovascular Society (CCS) class I to III will be included. By definition, CCS Grade IV (angina at rest for >20 min) is unstable angina [27], and these patients will not be recruited. We are recruiting participants from the University of Illinois Hospital in Chicago, Illinois. Participants are being recruited from the hospital, clinic, and through campus flyers (Figure 1).

The inclusion and exclusion criteria have been presented in Textboxes 1-3. Participants will continue to take their prescribed medications as directed. The study will be conducted in clinical research exam rooms. Licensed acupuncturists will administer 10 acupuncture treatments to 35 participants in the experimental group (and up to 34 participants in the control group upon study completion). We are using 7 acupuncturists to improve the likelihood that the protocol, and not the acupuncturist, is responsible for the therapeutic effects. All other therapeutic elements will be controlled through our standardized protocol. Acupuncturists will not discuss protocols with the participants.

**Screening tool—inclusion criteria.**

1. Confirmed diagnosis of stable angina for at least 6 months (pain, pressure, or discomfort in the chest or other areas of the upper body)
2. Experiencing symptoms at least once per week
3. Canadian Cardiovascular Society angina class I, II, III
4. Age 21 years or older
5. Speak and read English

**Screening tool—exclusion criteria.**

1. Physical or cognitive limitations that will prevent completion of study tasks (Mini-Cog score 0-2 with abnormal clock face);
2. Exacerbation of heart failure (B-type natriuretic peptide>500 or escalating dose of diuretic or admission to hospital for heart failure in past 3 months).
3. Autoimmune dysfunction (use of steroid or prescription anti-inflammatory medications)
4. Moderate to severe chronic obstructive pulmonary disease
  - Moderate (treated with short-acting bronchodilators plus antibiotics and/or oral corticosteroids)
  - Severe (patient requires hospitalization or visits the emergency room). Severe exacerbations may also be associated with acute respiratory failure
5. Pregnant
6. Use of prescription analgesic medications
7. Currently undergoing treatment with biofeedback, massage, or other acupuncture

**Screening tool—additional questions.**

1. Study explained (purpose, rights of participants, informed consent, compensation for time)
2. Has clear understanding of study purpose and requirements
3. Agrees to participate?
4. Reason for declining?
5. If declined: age, sex, race

### Measures

#### Demographic

Personal demographic characteristics (age, race/ethnicity, marital status, education, income, employment, and insurance) will be measured.

#### McGill Pain Questionnaire

The McGill Pain Questionnaire (MPQ) measures the multidimensionality of pain in 4 domains: sensory (location, intensity, quality, and pattern), affective (anxiety, depression, and fear), evaluative (a comprehensive assessment of the pain), and behavioral (behaviors that worsen or lessen pain) [28]. Current, least, and worst pain in the past 24 hours are scored on a Likert-type scale with 0=no pain to 10=excruciating pain. The MPQ has been well validated in cardiac populations [29-32].

#### Upper Body Diagram

Everts et al’s [33] upper body diagram consists of a drawing of the human chest with 9 distinct regions plus the neck, left and right arms, and abdomen. Participants are asked to mark the location of their pain on the diagram. The total pain sites are a location measure and will be used to calculate the multidimensionality of the participant’s pain.

#### Seattle Angina Questionnaire-7

The Seattle Angina Questionnaire-7 (SAQ-7) consists of 7 items in 4 domains (physical limitation, angina stability, angina frequency, and quality of life) measuring the impact of angina on participants’ health status. Item responses are coded sequentially from worst to best status and range from 1 to 6, except quality of life (range 1-5). Scores are generated for each domain and are scaled 0 to 100, with 0 denoting the worst and 100, the best possible status [34]. The SAQ-7 has been validated among patients with stable ischemic heart disease, those undergoing coronary interventions, and after acute myocardial infarction [35].

#### American Heart Association Angina Log

My Angina Log is a simple 1-page diary used to measure each episode of angina. Participants record the date and times they experienced angina, as well as triggers and treatments [36]. Severity of symptoms are measured on a 1 to 4 scale, with 1 representing mild symptoms and 4 very severe symptoms.

#### Protocol Acceptability Scale for Treating Angina With Acupuncture

This satisfaction instrument is a 10-item instrument used to measure acceptability of the study processes and protocols. Items are measured on a 0 (negative response; ie, did not like acupuncture) to 2 (positive response) scale. The protocol will be deemed to have high acceptability if 80% of participants score ≥16.

All data except for the American Heart Association (AHA) Angina log and the chest outline to draw angina pain will be collected via the Research and Electronic Data Capture (REDCap) application, which is a Web-based application for securely building and managing surveys and databases.

#### Biomarkers of Inflammation

Proinflammatory cytokines IL-1 beta, IL-2, IL-8, and IL-18; C-reactive protein (CRP) [37,38]; and TNF alpha, the anti-inflammatory cytokines IL-4 and IL-10 [37], and the dual pro/anti-inflammatory IL-6 [39] will be measured to determine if there is a significant change from baseline to post study (5 week). Venous blood (4.5 mL) will be collected in a blue-top collection tube containing 3.2% sodium citrate. Samples will be processed in our clinical lab. Samples will be centrifuged at room temperature for 15 min; plasma is then removed into 2 cryovials in 1 mL aliquots and stored in a –80°C freezer immediately. A label with study identification number, date, and time of blood draw will be placed on the cryovials before freezing. Samples will be batch analyzed using high-sensitivity multiplex technologies [40]. Table 1 lists all variables, measures, and data collection time points.

**Table 1:** Data collection variables, measures, and time points (both groups).

Variable		Measure/analyses	Data collection points		
			Baseline	x10	End
**Primary aim 1**					
	Recruitment	Number of enrolled/number invited to participate; Mini-Cog instrument (screening)	✓^a^	—^b^	—
	Retention	Number retained at study completion/number recruited	✓	—	✓
	Completion	Number of time points completed (11 total)	—	—	✓
	Acceptability	Protocol Acceptability Scale for Treating Angina with Acupuncture	—	—	✓
**Primary aim 2**					
	Demographic data	Demographic data form	✓	—	—
	Effect size	Intraindividual effect: mean prescores minus mean postscores/pooled SD. Group effect: mean differences in scores between acupuncture and attention control group	✓	—	✓
**Exploratory aim**					
	Biomarkers	Interleukin 1 beta, 2, 6, 8, 10, and 18; C-reactive protein, tumor necrosis factor alpha-α	✓	—	✓
**Outcome measures**					
	Pain and symptoms	McGill Pain Questionnaire, Seattle Angina Questionnaire-7, American Heart Association Angina Log	✓	✓	✓
	Functional status and HRQoL^c^	Seattle Angina Questionnaire-7, Physical Functioning and HRQoL subscales	✓	✓	✓

^a^Data were collected.

^b^Data were not collected.

^c^HRQoL: health-related quality of life.

### Randomization and Concealment

The research specialist will screen and consent eligible individuals (Textbox 1). Potential participants will be informed that they will be randomized to either the acupuncture group (10-acupuncture session protocol, 2 treatments per week for 5 weeks) or the attention control group (2-hour video session once a week for 5 weeks). After informed consent is obtained, participants will be randomized to the acupuncture or attention control group using the randomization module on REDCap [41] based on the stratified, permuted block schedule prepared by the biostatistician. We are concealing the order of treatment arm assignment from the research associate responsible for recruiting and randomizing through the use of REDCap’s restricted user rights. Our block sizes are concealed and our stratified approach also help to prevent research staff from guessing the sequence.

### Blinding

For this feasibility study, because of the unique nature of acupuncture and the difficulty in blinding participant and acupuncturist (see Study Design), acupuncturists and participants will not be blinded to the group assignment. Although it would be optimal to have the research associate who collects outcome data blinded, this is not possible for the current feasibility protocol. Instead, we are using all self-administered outcome measures collected on a tablet computer. The co-principal investigators (co-PIs) and the biostatistician will remain blinded to group membership for data analyses.

### Research Protocol

The research specialist will explain the nature of the study, risks, beneﬁts, the voluntary nature of participation, and the right to discontinue participation at any time without consequences. After informed consent is obtained, participants will be randomized to the acupuncture or attention control group. All participants complete 4 measures at baseline: the Demographic Questionnaire, the MPQ [42], the Upper Body Outline [33], and the SAQ-7 [34]. Members of the acupuncture and control groups repeat the measures at the beginning of each session, either all 10 acupuncture sessions or following the 5 video sessions. All participants complete the AHA Angina Log (diary of symptoms) throughout the study, whenever they have angina symptoms. We will make copies of their paper log each time they come to the clinic in case they misplace their logs. Venipuncture will be performed at baseline and study completion.

#### Acupuncture Intervention Protocol

The acupuncturist will swab each point with alcohol. Needles will be inserted using a standardized TCM point prescription and retained for 30 min. Each needle will be rotated 3 times to stimulate the *qi* in the meridian; 10 min after insertion, 20 min after insertion, and just before removal at 30 min. Needles will be inserted using the standards of clean needle technique established by the Council of Colleges of Acupuncture and Oriental Medicine [43]. One size acupuncture needle, 0.25 diameter × 40 mm length, will be used for all participants receiving acupuncture. All acupuncture needles are sterile, disposable, and made of surgical stainless steel with stainless steel wound heads. Sessions will be repeated twice weekly (with at least 2 off days in between) for 5 weeks (10 sessions).

#### Acupuncture Point Prescription for Angina

**Figure 2:**
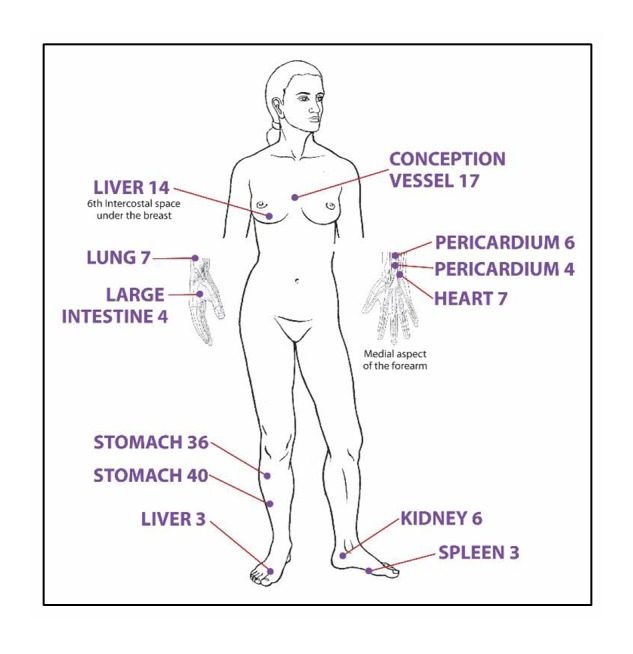
Acupuncture point prescription for angina. Used with permission from the Journal of Chinese Medicine Publications [17].

The standardized point prescription (Figure 2) uses acupuncture points on the front of the body to enable participants, many of whom are acupuncture-naïve, to remain supine. This is aimed at reducing anxiety by enabling the participant to anticipate needle insertions.

#### Attention Control Health Videos Protocol

The attention control group will watch health videos from the NOVA Science NOW series on Public Broadcasting System television. Topics do not contain content that could potentially improve pain. Videos are viewed from weeks 1 to 5 (5 time points) and equate to the time the experimental group receives acupuncture (10 hours) [44]. Titles include: “Can we live forever?,” Cracking your genetic code,” “Vaccines: Calling the shots,” “How does the brain work?,” “What are dreams?,” “How smart can we get?,” “Memory hackers, Cracking the code of life,” and “Can Alzheimer’s be stopped?.” The videos are shown in our private clinical research rooms. Standardized content assures complete fidelity to the control protocol.

### Retention Strategies

We anticipate an attrition rate of no more than 25%. These numbers are based on accruals and withdrawals in our previous studies and our detailed retention plan. Participants will be engaged as active partners in the research by addressing possible benefits of acupuncture (experimental group) and non–pain-related health education (control group), as well as the importance of their contribution to science. To minimize participant attrition and avoid missing data, we are (1) offering the control group the acupuncture protocol, free of charge, at the conclusion of the study; (2) scheduling data collection at convenient times for participants, including weekend and evening appointments; (3) contacting patients using their preferred mode of communication (phone, text, and email) before each appointment; (4) providing patients with study team contact information; and (5) providing patient honoraria (US $10 at each of 10 contact points for a total up to US $100).

### Data Safety Monitoring

The following data will be examined annually to evaluate safety issues every 6 months during the study: adverse events, attrition rate, reasons for attrition, and rate of adherence during the intervention. All adverse events will be monitored quarterly, but special attention will be given to serious adverse events that result in or require medical or surgical intervention to prevent death, a life-threatening situation, hospitalization, and a persistent or significant disability or incapacity. A Data and Safety Monitoring Committee (DMSC) has been appointed to provide oversight and monitoring of our data on an annual basis by individuals not directly associated with the study. The DMSC will consist of 2 scientists with expertise in RCT designs and a physician with expertise in the sample population. The co-PIs will meet with the DMSC annually and will present a written report as well as a verbal report. A report of the meeting will be submitted to the IRB. Additional meetings of the DMSC will be scheduled if the data are concerning or adverse events warrant more frequent review. If temporary or permanent suspension of the study occurs, the sponsoring institution IRBs and the National Institutes of Health will be notified.

### Adverse Events

No serious adverse events are anticipated in this low risk study; however, all adverse events (eg, hospitalization or injury) and/or unanticipated problems (eg, exacerbation of angina or other acute illnesses) will be reported in writing to the IRB at UIC within 5 days of discovery of the incident. Serious or unexpected adverse events will be verbally reported within 24 to 48 hours.

### Quality Assurance

Regular team meetings will be held for the duration of the research project, every 1 to 2 weeks. One principal investigator will chair these meetings. At the first several meetings, all recruitment and data collection procedures were reviewed with the research associate collecting data, and this process was and will be documented. To minimize potential for bias and to prevent contamination of the groups, 1 staff member will administer all instruments following acupuncture, and another staff member will administer all instruments following viewing of videos (for controls).

### Sample Size Calculation

We are powered to detect recruitment, retention, completion, and acceptability rates for a future efficacy study, with a 95% 2-sided CI. With our planned sample size of 69 patients, the margin of error will be 9.5% if feasibility rates are in the desired range of 80% or greater; however, will be a maximum of 11.8% if these rates are as low as 50% [45-47]. Similarly, we will compute a 2-sided 95% CI for our effect size estimates, with a half-width of 0.557 standard deviation for posttest differences between groups assuming completion by 75% of participants [48]. With a sample size of 52, we have 80% power to detect a large difference in mean change (0.7 standard deviation or greater) as statistically significant, assuming a repeated-measure correlation of 0.6 and a 2-sided *t* test with 0.05 significance level. We identified 1 similar study reporting continuous outcomes that found a large effect size of 0.85 for a pain reduction outcome and a 0.51 effect size for reduction in number of angina attacks per week [49].

### Statistical Analyses

Our biostatistician will supervise data management and data analysis procedures. All data will be exported from REDCap and imported into SAS version 9.4 for cleaning and analysis. Descriptive statistics (frequencies, means, and standard deviations) and bivariate statistics by treatment group will be used to describe the sample and assess baseline differences. Nonnormal distributions may be optimally transformed using the Box-Cox method and/or analyzed using a comparable nonparametric test. Specific plans for analysis are as follows:

- Aim 1: *To determine the feasibility (recruitment, completion,* and *acceptability rates of 80%; retention rate of 75%) of an RCT of acupuncture for stable angina*. We will calculate the proportion of participants who are recruited, retained in the study, and complete the protocol. We will also calculate a 95% CI for these estimates. Acceptability will be examined by proportion of participants (≥80%) scoring ≥16 on the acceptability instrument. These statistics will be used to determine if protocol changes are needed to conduct a larger multisite trial.
- Aim 2: *To estimate effect sizes for change from pre- to postintervention scores for pain/symptoms, inflammatory cytokines, functional status, and HRQoL between acupuncture and control groups.* Effect size estimates from the literature were not of high quality or sufficiently comparable with power for an efficacy trial; thus, we are estimating effect sizes. However, if effects are large, our well-designed study may provide preliminary efficacy for the acupuncture intervention. We will analyze the trial’s primary outcomes with a rigorous mixed-effect model for repeated measures recommended for primary analysis of clinical trials with continuous endpoints [50]. Biological sex will be included as a covariate. We will use intention-to-treat analyses, retaining all participants randomized to groups. Missing data will be addressed by using the full information maximum likelihood approach, which produces unbiased parameter estimates and SEs when data are missing at random [51,52]. Effect sizes and 95% Cis will be calculated for pain, and symptoms will be reported via survey and diary, functional status, and HRQoL. We will use the model parameter estimate for treatment group-by-time (baseline to end point), divided by the control group’s baseline standard deviation for a Glass delta effect size estimate using a bootstrap method for obtaining 95% CIs [53].
- Exploratory aim: *Explore between-group differences in levels of inflammatory biomarkers (IL-1 beta, IL- 2, IL-6, IL-8, IL-10,* and *IL-18; CRP; TNF alpha) from baseline to study completion (5 weeks).* We will calculate effect size estimates and CIs using mixed models for repeated measures models as stated in Aim 2. We will also explore how changes in biomarkers relate to change in self-reported pain. These exploratory analyses will inform mechanism hypotheses in a future trial.

### Ethical Considerations

The proposed protocol has only minimal potential physical or psychological risks to participants. The risks of this study are primarily those from acupuncture needle insertions including: soreness, minor bleeding, bruising after acupuncture needle removal, and fatigue after an acupuncture session. On the basis of findings from previous studies, the likelihood of these risks is very small [54]. There is also minor risk associated with venipuncture (for biomarkers). Collection of blood samples carry a small risk of pain or infection. Collection of data from questionnaires poses minimal time and emotional burden on participants. Finally, there may be some stress associated with keeping appointments for either the acupuncture intervention or the attention control video viewing session; this stress can be mitigated by emphasizing the voluntary nature of the visits, the expertise of the acupuncturist, and the comfortable and private surroundings.

Participants can refuse to participate in any or all of the study procedures or to withdraw from the study at any time. There are 3 categories of potential risk associated with this study: (1) Stress or emotional burden. Any psychological risks are likely to be related to items on the questionnaires that focus attention on symptoms and psychosocial variables. Participants may become uncomfortable thinking about their illness, treatment, or recovery. The major risk for this part of the study is the maintenance of confidentiality. Response burden for the participants is relatively low and will consist of time spent completing instruments, undergoing acupuncture, or watching health-related videos. If participants are tired or anxious, the sessions can be rescheduled for a later time; (2) Pain, discomfort, or physical harm. The risks from acupuncture needle insertions include soreness, minor bleeding, bruising after acupuncture needle removal, and fatigue after acupuncture. Blood will be obtained by venipuncture. Blood draws can cause syncope, temporary discomfort from the needle stick, bruising, and rarely infection. Patients will be encouraged to rest if necessary, and water and energy bars will be available for patients during each visit to the clinical research lab (for both groups); and (3) Loss of confidentiality. Every effort will be made to maintain patient privacy during data collection. In addition, a number of strategies to protect the privacy and confidentiality of each participant are in place including: proper and current Health Insurance Portability and Accountability Act (HIPAA) training and certification of all staff, compliance with HIPAA guidelines, storage of study data on a secure REDCap server and/or on a limited-access research drive, and placing angina logs and signed consent forms in a locked cabinet in the PIs office. All patients will participate only after written informed consent is obtained. The participants will retain a copy of the signed consent form. Guidelines for consent will be strictly followed according to the approval criteria of the IRB. All study data will be de-identified and stored in a private network drive at UIC that are accessible only to the co-PIs, biostatistician, and project director. Data will be collected on tablet computers, used solely for the study, and will be stored in a locked research office when not in use. Database access is limited to the co-PIs and the project director.

## Results

This study has been funded over 2 years by the National Institutes of Health, National Institute for Nursing Research. We are currently recruiting and expect to have initial results by December 2020.

## Discussion

### Overview

The incidence of stable angina is projected to rise up to 18%, with concomitant increases in comorbid conditions [55]. It is well known that angina patients complain of side effects from antianginal drugs, including nitroglycerin-related headaches, isosorbide-related dizziness and nausea/vomiting, and beta-blocker-related fatigue, nightmares, and depression which may lead to nonadherence and increased risk for future cardiac events [5,56] A reduction in pain and associated symptoms of stable angina has the potential to improve functional status and HRQoL [3]. After beginning efficacy of this acupuncture protocol is established, future studies will include determining acupuncture from placebo effect, duration of effect, and optimal dosage effect [24,57]. These findings will then provide insight into the need for maintenance acupuncture treatments to reduce the chronic pain of stable angina.

### Conclusions

This protocol represents the first step in examining acupuncture as a nonpharmacologic approach to the treatment of symptoms associated with stable angina. Acupuncture has been shown to have no major side effects [54,57]. Demonstrating the beginning efficacy of acupuncture for the reduction of symptoms of stable angina may shift its treatment from a purely Western model to a complementary model of care.

## Appendix

Multimedia Appendix 1 Peer-reviewer report from the National Institutes of Health, National Institute of Nursing Research.

## Abbreviations

AHA: American Heart Association
CCS: Canadian Cardiovascular Society
Co-PI: co-principal investigator
CRP: C-reactive protein
DMSC: Data and Safety Monitoring Committee
HIPAA: Health Insurance Portability and Accountability Act
HRQoL: health-related quality of life
IL: interleukin
IRB: Institutional Review Board
MPQ: McGill Pain Questionnaire
RCT: randomized controlled trial
SAQ-7: Seattle Angina Questionnaire-7
TCM: traditional Chinese medicine
TNF: tumor necrosis factor
UIC: University of Illinois at Chicago
