# Supplementary material for: Acupuncture to Improve Symptoms for Stable Angina: Protocol for a Randomized Controlled Trial
Source: JMIR Res Protoc. 2019 Jul 29;8(7):e14705. doi: 10.2196/14705 (PMC6690225; doi:10.2196/14705)
Supplement: Multimedia Appendix 1 [file resprot_v8i7e14705_app1.pdf]

SUMMARY STATEMENT

PROGRAM CONTACT:
Lois Tully
301-594-5968
tullyla@mail.nih.gov

(Privileged Communication)

Release Date: 06/26/2018
Revised Date:

Application Number: 1 R21 NR017705-01A1

Principal Investigators (Listed Alphabetically):

DEVON, HOLLI A (Contact)
SCHLAEGER, JUDITH MICHELLE

Applicant Organization: UNIVERSITY OF ILLINOIS AT CHICAGO

Review Group: NRCS
Nursing and Related Clinical Sciences Study Section

Meeting Date: 06/07/2018
Council: OCT 2018
Requested Start: 09/01/2018
RFA/PA: PA18-156
PCC: BSMLT

Project Title: Feasibility Testing of a Randomized Controlled Trial of Acupuncture to Improve Symptoms for Stable Angina (AIMS-A)
SRG Action: Impact Score:18
Next Steps: Visit https://grants.nih.gov/grants/next_steps.htm
Human Subjects: 30-Human subjects involved - Certified, no SRG concerns
Animal Subjects: 10-No live vertebrate animals involved for competing appl.
Gender: 1A-Both genders, scientifically acceptable
Minority: 1A-Minorities and non-minorities, scientifically acceptable
Children: 3A-No children included, scientifically acceptable

Project Year	Direct Costs Requested	Estimated Total Cost
1	150,000	239,850
2	125,000	199,875
TOTAL	275,000	439,725

ADMINISTRATIVE BUDGET NOTE: The budget shown is the requested budget and has not been adjusted to reflect any recommendations made by reviewers. If an award is planned, the costs will be calculated by Institute grants management staff based on the recommendations outlined below in the COMMITTEE BUDGET RECOMMENDATIONS section.

1R21NR017705-01A1 DEVON, HOLLI

RESUME AND SUMMARY OF DISCUSSION: This application proposes a randomized attention-control trial to test the feasibility and preliminary efficacy of acupuncture therapy for patients with symptomatic stable angina. The proposed study is highly significant and if successful, findings will inform complementary approaches to conventional medical treatment for improving symptom management in this population. This is a strong multidisciplinary team with complementary skills needed to carry out the proposed study. The project is strengthened by changes that are very responsive to concerns identified in the previous review, including the use of two acupuncturists and an improved power analysis. The premise upon which the study is based is well-supported by the extant literature. The design is scientifically rigorous and well-developed; notable strengths include a well-considered attention control group and retention plan. Detracting only slightly from these strong points are some concerns related to a lack of specification of the primary outcome for the trial and no clinical criteria for baseline angina pain severity but these were considered minor and detract only minimally from this otherwise compelling project. Overall, the panel agreed that findings are expected to have a high impact on personalized strategies to manage symptoms of chronic illness.

DESCRIPTION (provided by applicant): Despite a notable 32.7% decline in mortality from ischemic heart disease (IHD) since 1999, the prevalence of stable angina has not decreased. Stable angina is defined as predictable chest pain on exertion or under mental or emotional stress. Stable angina signifies partial obstruction of coronary flow or microvascular changes and comes with substantial lifetime consequences including heart failure, atrial fibrillation, reinfarction, and cardiac arrest. Nearly 9 million Americans have stable angina and heart disease is now the leading disability-adjusted disease globally. Stable angina is associated with multiple symptoms, impaired functional status, and reduced health-related quality of life (HRQoL). The aims of this randomized controlled trial are to test the feasibility and preliminary efficacy of acupuncture therapy for patients with stable angina and to compute effect sizes for between group differences in pre- and post-test pain, associated symptoms, inflammatory biomarkers, functional status, and HRQoL. Sixty-nine patients hospitalized with IHD and experiencing symptoms of stable angina will be recruited from an academic medical center. Those with a confirmed diagnosis of stable angina, and treated with medical therapy for at least 6 months, will be randomly assigned to the acupuncture or attention control group. Participants in the acupuncture group will receive a standardized Traditional Chinese Medicine (TCM) point prescription. Acupuncture will be administered 2 times per week for 5 weeks for a total of 10 treatments. The attention control group will view non-pain related health education videos over 5 weeks equal to the 10 hours of treatment for the acupuncture group. Upon completion of the study, the attention control group will be offered the same acupuncture protocol as the intervention group. The McGill Pain Questionnaire and the American Heart Association (AHA) Angina Log will be used to measure pain location, intensity, quality, pattern and associated symptoms. The Seattle Angina Questionnaire- 7 will be used to measure symptoms other than pain, functional status, and HRQoL. Participants will complete measures of anginal pain (average pain compared to; pain now, worst pain, & least pain in the past 24 hours) and the AHA Angina Log before each acupuncture treatment. Average pain and “pain now” will be measured after each treatment. Outcome measures for recruitment, retention, completion of data, and patient acceptability will be measured at the conclusion of the study. Feasibility, defined as recruitment and retention rates >75%, and acceptability (>80% acceptable) will be analyzed using descriptive statistics. Effect sizes will be computed for differences in pre- and post-test pain, symptoms, and HRQoL. The outcome variables of pain, symptoms, functional status, and HRQoL will be analyzed using mixed regression models to determine if there are significant benefits of acupuncture if effect sizes are large. Symptom management is critical for reducing disability and improving HRQoL for those with angina.

PUBLIC HEALTH RELEVANCE: The outcome of this novel study will be to advance science by providing new data, in an American population, on the feasibility of a trial of acupuncture for the treatment of symptomatic stable angina. This study also has the potential to improve patient care and

patient outcomes since our results may suggest that acupuncture is efficacious as a complementary therapy for women and men with stable angina thus improving symptom management.

CRITIQUE 1

Significance: 1
Investigator(s): 2
Innovation: 2
Approach: 3
Environment: 1

Overall Impact: This is an R21 application submitted by multi-PIs Drs. Devon and Schlaeger to test the feasibility of acupuncture for management of stable angina. The researchers have been responsive to prior reviewers' feedback. Stable angina affects nine million Americans. Pain and symptom control is of clinical and scientific significance in this population. Acupuncture, a popular CAM approach, has some promising effects for managing angina in Chinese literature; however, its investigation in the US is extremely limited. Therefore, this application is innovative because it tests the feasibility of acupuncture as a complementary approach in addition to conventional medical treatment. Drs. Devon and Schlaeger are excellent nursing scientists who have assembled a multidisciplinary team of investigators with expertise in cardiology, nursing, acupuncture, statistics, and biomarker analyses. The study design has numerous strengths including randomized trial, attention control, and a careful retention plan. A few addressable limitations include: lack of specification of a primary outcome for the trial and no inclusion criteria for the severity of symptoms of stable criteria. UIC's academic and clinical environment is excellent for supporting the proposed application. Overall this application has excellent to outstanding impact.

1. Significance:

Strengths

- Stable angina affects nearly nine million Americans.
- Acupuncture has some potential effects based on literature in Chinese population; however, very little research has been conducted in the US.
- If successful, this and future studies can improve pain management for Americans experience angina pain.

Weaknesses

- Lack of description of what acupuncture would be expected to improve as angina pain can be episodic and brought on by activities/exertion. Is the pain severity to be improved or episodes of angina to be reduced? There is no clear description from the proposed trial.

2. Investigator(s):

Strengths

- This study would benefit from a multiple PI structure. Both Drs. Devon and Schlaeger are outstanding nursing scientists who bring complementary expertise.
- Dr. Devon has considerable expertise in cardiovascular health.
- Dr. Schlaeger has expertise in acupuncture and pain research.
- Dr. Steffen brings her statistical expertise to the trial.
- Dr. Shroff brings his medical expertise in cardiology.

- Dr. Hoppensteadt will help with analyses of biomarkers.

Weaknesses

- None.

3. Innovation:

Strengths

- This is the first study in the US to evaluate the feasibility of acupuncture for stable angina.

Weaknesses

- None.

4. Approach:

Strengths

- Randomized controlled trial.
- Carefully developed acupuncture protocol by a knowledgeable nursing scientist who is also an acupuncturist.
- Attention control choice was justified.
- Thoughtful recruitment and retention plan.

Weaknesses

- No primary outcome was specified for a clinical trial (Pain severity? Episodes of pain?)
- No clinical criteria for baseline angina pain severity was chosen.

5. Environment:

Strengths

- The UIC College of Nursing has outstanding resources.
- Letters from the UIC Hospital CEO and cardiology collaborator are very supportive.

Weaknesses

- None.

Study Timeline:

Strengths

- The timeline for key activities were outlined.

Weaknesses

- A more specific accrual target (i.e. recruitment per month) will be helpful to plan for successful trial conduct.

Protections for Human Subjects:

Acceptable Risks and/or Adequate Protections

- No concerns were identified.

Data and Safety Monitoring Plan (Applicable for Clinical Trials Only):

Acceptable

- No concerns were identified.

Inclusion of Women, Minorities and Children:

- Sex/Gender: Distribution justified scientifically
- Race/Ethnicity: Distribution justified scientifically
- For NIH-Defined Phase III trials, Plans for valid design and analysis: Not applicable
- Inclusion/Exclusion of Children under 18: Excluding ages <18; justified scientifically

Vertebrate Animals:

Not Applicable (No Vertebrate Animals)

Biohazards:

Not Applicable (No Biohazards)

Resubmission:

- No concerns were identified.

Resource Sharing Plans:

Acceptable

Authentication of Key Biological and/or Chemical Resources:

Acceptable

Budget and Period of Support:

Recommend as Requested

CRITIQUE 2

Significance: 2
Investigator(s): 2
Innovation: 2
Approach: 2
Environment: 2

Overall Impact: This proposal is to conduct a randomized clinical trial (RCT) of acupuncture vs control to reduce pain and symptoms in patients with stable angina. The main objective is to estimate effect sizes to inform the design of larger RCTs. The study is unique in that such studies have not been carried out in American populations and that data on inflammatory markers will be collected to address mechanisms of action. The trial is clearly described and is likely to have moderate to high impact.

1. Significance:

Strengths

- Stable angina is a significant cardiovascular health problem, affecting 9 million Americans.
- Stable angina is associated with multiple symptoms, impaired functional status, and reduced health-related quality of life (HRQoL).
- A reduction in pain and associated symptoms has the potential to improve functional status and HRQoL.
- Acupuncture has potential analgesic and anti-inflammatory effects, which might make it a suitable treatment for angina.

Weaknesses

- None noted.

2. Investigator(s):

Strengths

- Statistical support will be provided by Alana Steffen, PhD, Assistant Professor (0.6 CM Years 1 & 2). Although not formally trained as a statistician, her CV demonstrates that she is qualified to provide this support.

Weaknesses

- None noted

3. Innovation:

Strengths

- The use of acupuncture (a CAM modality) to help control angina pain is novel for American populations.
- Evaluation of inflammatory markers may shed light on the mechanisms involved in angina pain control using acupuncture.

Weaknesses

- None noted.

4. Approach:

Strengths

- Randomized trial with attention control group.
- Attention control group will eventually be offered acupuncture. This should reduce the dropout rate in the control group.
- Methods, procedures, and outcome measures are well-described. Proposed statistical methods are appropriate.
- Applicant has satisfactorily responded to the previous (statistical) review by adding a second acupuncturist (for increased generalizability) and by reducing the retention rate criterion from 80% to 75%.
- Although there is no discussion of sex as a biological variable, investigators will be using sex as a stratification variable prior to randomization.

Weaknesses

- To inform future RCTs that will employ intention-to-treat (ITT), it would be helpful to use the proposed study to estimate the proportion of acupuncture dropouts who return for completion of the McGill and SAQ-7.

5. Environment:

Strengths

- Environment will adequately support the proposed research.

Weaknesses

- None noted.

Study Timeline:

Strengths

- Realistic.

Weaknesses

- None noted.

Protections for Human Subjects:

Acceptable Risks and/or Adequate Protections

- No concerns were identified.

Data and Safety Monitoring Plan (Applicable for Clinical Trials Only):

Acceptable

Inclusion of Women, Minorities and Children:

- Sex/Gender: Distribution justified scientifically
- Race/Ethnicity: Distribution justified scientifically
- For NIH-Defined Phase III trials, Plans for valid design and analysis: Scientifically acceptable
- Inclusion/Exclusion of Children under 18: Excluding ages <18; justified scientifically

Vertebrate Animals:

Not Applicable (No Vertebrate Animals)

Biohazards:

Not Applicable (No Biohazards)

Resubmission:

- No concerns were identified.

Resource Sharing Plans:

Acceptable

Authentication of Key Biological and/or Chemical Resources:

Not Applicable

Budget and Period of Support:

Recommend as Requested

CRITIQUE 3

Significance: 2
Investigator(s): 1
Innovation: 1
Approach: 2
Environment: 1

Overall Impact: In this R21 application, the investigators will test the feasibility and preliminary efficacy of acupuncture in a sample of individuals with stable angina randomized to receive acupuncture or attention control health education. The overall level of impact is high as there are few methods to manage pain associated with angina that are underpinned by a biological mechanism, inflammation and blood flow. However, the specific relationships between blood flow and inflammation have yet to be determined. There are numerous score driving strengths including a strong scientific premise based on a lack of research specific to acupuncture as a complementary treatment for angina aimed at U.S. populations, specifically women who experience more microvascular angina. The team of complementary experts is highly qualified to conduct the study, there are several innovations such as inclusion of biomarkers and a theoretical acupuncture model, and approaches that are rigorous (multiple measures of pain, delivery of the intervention by two acupuncturists, having attention control group that will be offered the treatment after the study), and a very supportive environment. The investigators have been responsive to the prior critique. If the findings indicate feasibility of the study procedures and signals of efficacy, these data will provide evidence to support an adequately powered RCT. There is a high likelihood that the proposed research will have a sustained and powerful influence on the field of pain management, and cardiovascular clinical care specific to symptom science.

1. Significance:

Strengths

- This group experiences frequent, high level pain that leads to substantial disability and poor quality of life.
- There is a strong scientific premise - there are low quality data on outcomes associated with symptom reduction in patients with angina, non-adherence to medications is common due to side effects, and there is a large body of knowledge that acupuncture has demonstrated physiologic analgesic effects.
- Preliminary research conducted by the team will be applied to this understudied population for which few effective therapies are available for symptom management.
- There is high potential to advance scientific understanding of acupuncture in a cardiac population which could be very translatable to other chronic cardiac conditions where there is high symptom burden including pain.

Weaknesses

- None noted.

2. Investigator(s):

Strengths

- Dr. Devon and her team are highly experience and well-established investigators and are well suited to conduct the project.
- Dr. Devon has conducted multiple clinical trials and has the expertise to lead the team in coordination and data management.
- Dr. Schlaeger, the co-PI, developed an acupuncture point prescription for treating stable angina. This will allow for standardization of the study acupuncture procedures.

Weaknesses

- None noted.

3. Innovation:

Strengths

- While this is a feasibility study, there is high likelihood that findings could shift clinical practice by adding a new approach to complementary manage angina based on inflammatory pathways.
- The Theory of Traditional Chinese Medicine is used to guide blood flow balance and thus may have a direct influence on inflammation.
- The protocol will be tested in a sample of Americans with stable angina who have not been previously tested in the U.S.

Weaknesses

- None noted.

4. Approach:

Strengths

- Sex as a biological variable is addressed as there are differences in the pathophysiology of disease in women who have poorer outcomes compared to men. The sample will be stratified by biological sex providing a balanced distribution.
- The acupuncture treatment is well described and will be delivered by two acupuncturists.
- Feasibility measures are appropriate and include a validated measure of acceptability specific to acupuncture.

Weaknesses

- There is a lack of detail regarding fidelity monitoring of the treatment delivery during acupuncture sessions.

5. Environment:

Strengths

- College of Nursing at the University of Illinois at Chicago has the resources for conducting the trial (exam rooms) and laboratory support to analyze the specimens.
- There is a large cadre of patients at the medical center will provide an adequate sample of underrepresented Blacks and Hispanics from which to recruit.

Weaknesses

- None noted.

Study Timeline:

Strengths

- The timeline considers the justification of the timelines; potential challenges and solutions are provided that are realistic. Anticipate start date is April 2019.

Weaknesses

- None noted.

Study Timeline:

Strengths

- No concerns were identified.

Weaknesses

- None noted.

Protections for Human Subjects:

Acceptable Risks and/or Adequate Protections

- Minimal risk study with well described human subject's protections.

Data and Safety Monitoring Plan (Applicable for Clinical Trials Only):

Acceptable

- Committee will be appointed and the process for reporting adverse events is outlined.

Inclusion of Women, Minorities and Children:

- Sex/Gender: Distribution justified scientifically
- Race/Ethnicity: Distribution justified scientifically
- For NIH-Defined Phase III trials, Plans for valid design and analysis: Not applicable
- Inclusion/Exclusion of Children under 18: Excluding ages <18; justified scientifically
- There is no rationale for why individuals 18 to 21 years of age will not be included in the study. The investigators do note that in previous studies the age range was 29 - 98 years. Minorities will comprise 55% of the sample.

Vertebrate Animals:

Not Applicable (No Vertebrate Animals)

Biohazards:

Not Applicable (No Biohazards)

Resubmission:

- The investigators have adequately addressed key concerns noted in the previous critique, specifically the use of two acupuncturists, clarification of the power analysis, and adding Dr. Schlaeger as a co-PI who has a large role in the study as an interventionist.

Resource Sharing Plans:

Acceptable

Authentication of Key Biological and/or Chemical Resources:

Not Applicable (No Relevant Resources)

Budget and Period of Support:

Recommend as Requested

- Effort for the PI and Co-PI is in line with roles and responsibilities.

THE FOLLOWING SECTIONS WERE PREPARED BY THE SCIENTIFIC REVIEW OFFICER TO SUMMARIZE THE OUTCOME OF DISCUSSIONS OF THE REVIEW COMMITTEE, OR REVIEWERS' WRITTEN CRITIQUES, ON THE FOLLOWING ISSUES:

PROTECTION OF HUMAN SUBJECTS: ACCEPTABLE

INCLUSION OF WOMEN PLAN: ACCEPTABLE

INCLUSION OF MINORITIES PLAN: ACCEPTABLE

INCLUSION OF CHILDREN PLAN: ACCEPTABLE

COMMITTEE BUDGET RECOMMENDATIONS: The budget was recommended as requested.

Footnotes for 1 R21 NR017705-01A1; PI Name: DEVON, HOLLI A

NIH has modified its policy regarding the receipt of resubmissions (amended applications). See Guide Notice NOT-OD-14-074 at http://grants.nih.gov/grants/guide/notice-files/NOT-OD-14-074.html. The impact/priority score is calculated after discussion of an application by averaging the overall scores (1-9) given by all voting reviewers on the committee and multiplying by 10. The criterion scores are submitted prior to the meeting by the individual reviewers assigned to an application, and are not discussed specifically at the review meeting or calculated into the overall impact score. Some applications also receive a percentile ranking. For details on the review process, see http://grants.nih.gov/grants/peer_review_process.htm#scoring.

MEETING ROSTER

Nursing and Related Clinical Sciences Study Section
Healthcare Delivery and Methodologies Integrated Review Group
CENTER FOR SCIENTIFIC REVIEW
NRCS
06/07/2018 - 06/08/2018

Notice of NIH Policy to All Applicants: Meeting rosters are provided for information purposes only. Applicant investigators and institutional officials must not communicate directly with study section members about an application before or after the review. Failure to observe this policy will create a serious breach of integrity in the peer review process, and may lead to actions outlined in NOT-OD-14-073 at https://grants.nih.gov/grants/guide/notice-files/NOT-OD-14-073.html and NOT-OD-15-106 at https://grants.nih.gov/grants/guide/notice-files/NOT-OD-15-106.html, including removal of the application from immediate review.

CHAIRPERSON(S)

SOLE, MARY LOU, PHD
DEAN AND PROFESSOR
COLLEGE OF NURSING
UNIVERSITY OF CENTRAL FLORIDA
ORLANDO, FL 32826

MEMBERS

ALBRECHT, TARA A., BSN, PHD *
ASSISTANT PROFESSOR
SUSAN WHITE HOLSWORTH PALLIATIVE CARE SCHOLAR
DIVISION OF HEMATOLOGY/ONCOLOGY
DEPARTMENT OF INTERNAL MEDICINE
VIRGINIA COMMONWEALTH UNIVERSITY
RICHMOND, VA 23298

AOUIZERAT, BRADLEY E, MS, PHD
PROFESSOR AND DEPUTY DIRECTOR
DEPARTMENT OF ORAL AND MAXILLOFACIAL SURGERY
BLUESTONE CENTER FOR CLINICAL RESEARCH
NEW YORK UNIVERSITY
NEW YORK, NY 10010

BAKITAS, MARIE ANNE, DNSC
PROFESSOR
MARIE O'KOREN ENDOWED CHAIR
SCHOOL OF NURSING
CENTER FOR PALLIATIVE AND SUPPORTIVE CARE
UNIVERSITY OF ALABAMA AT BIRMINGHAM
BIRMINGHAM, AL 35294

BURGENER, SANDRA C., FAAN, PHD, RN *
ASSOCIATE PROFESSOR EMERITA
DEPARTMENT OF BIOBEHAVIORAL HEALTH NURSING
UNIVERSITY OF ILLINOIS AT URBANA-CHAMPAIGN
URBANA, IL 46202

CHANG, CHIH-HUNG, PHD *
PROFESSOR
BUEHLER CENTER ON AGING, HEALTH AND SOCIETY
INSTITUTE FOR PUBLIC HEALTH MEDICINE
FEINBERG SCHOOL OF MEDICINE
NORTHWESTERN UNIVERSITY
CHICAGO, IL 60611

CORWIN, ELIZABETH JEANNE, PHD
PROFESSOR AND ASSOCIATE DEAN FOR RESEARCH
SCHOOL OF NURSING
EMORY UNIVERSITY
ATLANTA, GA 30322

DONOVAN, HEIDI, PHD *
PROFESSOR
VICE CHAIR FOR RESEARCH
DEPARTMENT OF HEALTH AND COMMUNITY SYSTEMS
SCHOOL OF NURSING
UNIVERSITY OF PITTSBURGH
PITTSBURGH, PA 15261

EDLUND, MARK J., MD, PHD *
SENIOR RESEARCH SCIENTIST
BEHAVIORAL HEALTH EPIDEMIOLOGY PROGRAM
RTI INTERNATIONAL
RESEARCH TRIANGLE PARK, NC 27709

ERSEK, MARY, BSN, PHD *
PROFESSOR
BIOBEHAVIORAL HEALTH SCIENCES
SCHOOL OF NURSING
UNIVERSITY OF PENNSYLVANIA
PHILADELPHIA, PA 19104

GROER, MAUREEN EDITH, FAAN, PHD, RN *
PROFESSOR
COLLEGE OF NURSING
UNIVERSITY OF SOUTH FLORIDA
TAMPA, FL 33612

HAMPSTEAD, BENJAMIN MICHAEL, PHD *
ASSOCIATE PROFESSOR
DEPARTMENT OF PSYCHIATRY
MICHIGAN ALZHEIMER DISEASE CENTER
UNIVERSITY OF MICHIGAN
ANN ARBOR, MI 48105

HANSEN, MATTHEW LEE, MD *
ASSOCIATE PROFESSOR
DEPARTMENT OF EMERGENCY MEDICINE
OREGON HEALTH & SCIENCE UNIVERSITY
PORTLAND, OR 97239

HICKMAN, RONALD LEE JR, FAAN, PHD, RN *
ASSOCIATE PROFESSOR
FRANCES PAYNE BOLTON SCHOOL OF NURSING
CASE WESTERN RESERVE UNIVERSITY
CLEVELAND, OH 44118

HOCKENBERRY, MARILYN J, FAAN, RN, PHD
ASSOCIATE DEAN FOR RESEARCH
BESSIE BAKER PROFESSOR OF NURSING
PROFESSOR OF PEDIATRICS
SCHOOL OF NURSING
DUKE UNIVERSITY
DURHAM , NC 27710

HOLLENBEAK, CHRISTOPHER S, PHD *
PROFESSOR
DEPARTMENT OF HEALTH POLICY AND ADMINISTRATION
PENNSYLVANIA STATE UNIVERSITY
UNIVERSITY PARK, PA 16802

HUPCEY, JUDITH E, EDD, FAAN
PROFESSOR AND ASSOCIATE DEAN FOR GRADUATE
EDUCATION AND RESEARCH
COLLEGE OF NURSING
PENNSYLVANIA STATE UNIVERSITY
HERSHEY, PA 17033

HUR, CHIN, MD *
ASSOCIATE PROFESSOR
DEPARTMENT OF MEDICINE
HARVARD MEDICAL CENTER
MASSACHUSETTS GENERAL HOSPITAL
BOSTON, MA 02114

JATOI, AMINAH, MD *
PROFESSOR
DEPARTMENT OF ONCOLOGY
COLLEGE OF MEDICINE
MAYO CLINIC
ROCHESTER, MN 55905

KELECHI, TERESA J, PHD
PROFESSOR
COLLEGE OF NURSING
MEDICAL UNIVERSITY OF SOUTH CAROLINA
CHARLESTON, SC 29425

KENDRICK, JESSICA B, MD *
ASSOCIATE PROFESSOR
UNIVERSITY OF COLORADO SCHOOL OF MEDICINE
DENVER HEALTH MEDICAL CENTER
DIVISION OF RENAL DISEASES AND HYPERTENSION
DENVER, CO 80204

LALLY, ROBIN M, BSN, PHD *
PROFESSOR
CENTER FOR NURSING SCIENCE
COLLEGE OF NURSING
UNIVERSITY OF NEBRASKA MEDICAL CENTER
OMAHA, NE 68198

LEE, KATHRYN, PHD, RN *
PROFESSOR AND JAMES P AND MARJORIE A LIVINGSTON
ENDOWED CHAIR IN NURSING
SYMPTOM MANAGEMENT
FAMILY HEALTH CARE NURSING
UNIVERSITY OF CALIFORNIA SAN FRANCISCO
SAN FRANCISCO, CA 94143

LESSER, MARTIN L., PHD *
PROFESSOR AND DIRECTOR
DIRECTOR AND INVESTIGATOR, BIOSTATISTICS UNIT
FEINSTEIN INSTITUTE FOR MEDICAL RESEARCH
MANHASSET, NY 11030

LINCOLN, JOHN A., MD, PHD *
ASSOCIATE PROFESSOR
DEPARTMENT OF NEUROLOGY
MCGOVERN MEDICAL SCHOOL
THE UNIVERSITY OF TEXAS
HEALTH SCIENCE CENTER AT HOUSTON
HOUSTON, TX 77030

MAO, JUN J, MD *
LAURANCE S. ROCKEFELLER CHAIR
IN INTEGRATIVE MEDICINE
MEMORIAL SLOAN KETTERING CANCER CENTER
BENDHEIM INTEGRATIVE MEDICINE CENTER
NEW YORK, NY 10021

MITCHELL, PAMELA H, BSN, PHD *
PROFESSOR
BEIOBEHAVIORAL NURSING
AND HEALTH INFORMATICS
DEPARTMENT OF HEALTH SERVICES
UNIVERSITY OF WASHINGTON
SEATTLE, WA 98195

PATEL, ALOKA LAHOTI, MD *
ASSOCIATE PROFESSOR
DEPARTMENT OF PEDIATRICS
RUSH UNIVERSITY MEDICAL SCHOOL
CHICAGO, IL 60612

RUBINSTEIN, ISRAEL, MD, PHD
PROFESSOR
DEPARTMENT OF MEDICINE
JESSE BROWN VA MEDICAL CENTER,
R AND D SERVICE
UNIVERSITY OF ILLINOIS AT CHICAGO
CHICAGO, IL 60612

SCHROECK, FLORIAN R, MD *
SECTION CHIEF OF UROLOGY
ASSISTANT PROFESSOR OF SURGERY
WHITE RIVER JUNCTION VETERANS AFFAIRS MEDICAL CTR
DARTMOUTH HITCHCOCK MEDICAL CENTER
LEBANON, NH 03756

SENATHIRAJAH, YALINI, PHD *
VISITING ASSOCIATE PROFESSOR
DEPARTMENT OF BIOMEDICAL INFORMATICS
SCHOOL OF MEDICINE
UNIVERSITY OF PITTSBURGH
PITTSBURGH, PA 15206

SINGER, JONATHAN PAUL, MD *
ASSISTANT PROFESSOR
DEPARTMENT OF MEDICINE
SCHOOL OF MEDICINE
UNIVERSITY OF CALIFORNIA, SAN FRANCISCO
SAN FRANCISCO, CA 94117

STONE, PATRICIA W, RN, PHD
CENTENNIAL PROFESSOR OF HEALTH POLICY
SCHOOL OF NURSING
COLUMBIA UNIVERSITY
NEW YORK, NY 10032

SUMAN, OSCAR E, PHD
PROFESSOR
DEPARTMENT OF SURGERY
SCHOOL OF MEDICINE
UNIVERSITY OF TEXAS MEDICAL BRANCH
GALVESTON, TX 77550

WILKIE, DIANA J, BSN, FAAN, PHD, RN *
PRAIRIEVIEW TRUST-EARL AND MARGO POWERS
ENDOWED PROFESSOR
COLLEGE OF NURSING
UNIVERSITY OF FLORIDA
GAINESVILLE, FL 32610

MAIL REVIEWER(S)

PANDOLFINO, JOHN E, MD
PROFESSOR
DIVISION OF GASTROENTEROLOGY
DEPARTMENT OF MEDICINE
FEINBERG SCHOOL OF MEDICINE
NORTHWESTERN UNIVERSITY
CHICAGO, IL 60611

TRAVAGLI, RENATO ALBERTO, PHD
PROFESSOR
DEPARTMENT OF NEURAL AND BEHAVIORAL SCIENCES
COLLEGE OF MEDICINE
PENNSYLVANIA STATE UNIVERSITY
HERSHEY, PA 17033

SCIENTIFIC REVIEW OFFICER

HARE, MARTHA L, PHD
SCIENTIFIC REVIEW OFFICER
CENTER FOR SCIENTIFIC REVIEW
NATIONAL INSTITUTES OF HEALTH
BETHESDA, MD 20892

EXTRAMURAL SUPPORT ASSISTANT

JONES, BELINDA
EXTRAMURAL SUPPORT ASSISTANT
CENTER FOR SCIENTIFIC REVIEW
NATIONAL INSTITUTES OF HEALTH
BETHESDA, MD 20892

OTHER REVIEW STAFF

TINKER, REBECCA I, PHD
SCIENTIFIC REVIEW STAFF (CONTRACTOR)
CENTER FOR SCIENTIFIC REVIEW
NATIONAL INSTITUTES OF HEALTH
BETHESDA, MD 20817

* Temporary Member. For grant applications, temporary members may participate in the entire meeting or may review only selected applications as needed.

Consultants are required to absent themselves from the room during the review of any application if their presence would constitute or appear to constitute a conflict of interest.
